# A Medical Causerie

**Published:** 1915-05-29

**Authors:** 


### A MEDICAL CAUSERIE.

The anti-inoculation campaign has not been a brilliant one, nor has it engaged much popular interest. Naturally it received the ardent sympathies of the anti-vaccinators and the anti-vivisectionists. One of the leading prophets among the latter wrote to the *Times* denouncing Sir Almroth Wright, but apparently no one heeded him. More sad still, we now learn that there are editors even more hard-hearted than those of Printing House Square, for a medical anti-vaccinationist tells us that he has written hundreds of letters to various newspapers, and not half a dozen of them have escaped the fate which, in the shape of a basket, lurks ever at the editorial elbow. Even the gentleman whose letters on the war are more popular in Germany than in his own country seems to be neglected, and the orators and handbills are wasted efforts. So conspicuously is this the case that the Government has decided to treat the whole matter with contempt, as an answer given last week in the House of Commons plainly shows. A great European war and a fight for national existence do not repress the agitator, but they throw him into the background and he finds the position an uncongenial one. In due time, no doubt, we shall find him as frank, candid, accurate, and modest as of yore, but for the present his antics fail to catch the public eye. When the country is saved he may come into his own again, and we shall do our best to suffer him gladly.

There are, however, exceptions to every rule, and one of our *confrères* has felt that the passive policy and the Apostolic injunction need modification. Perhaps the return of the warm weather is in part responsible. Anyhow, a well-known aural surgeon, armed with a walking-stick, seeing the display on the walls of a drapery establishment in the West End of posters warning recruits on the subject of inoculation, was moved to the attack and proceeded to tear down the obnoxious announcement. Argument followed between the surgeon and some members of the drapery staff, but this attempt at diplomacy was brusquely interrupted by the proprietor who, approaching from behind, delivered by means of an umbrella a counter-attack on the surgical headgear. Thereupon, as the rules of warfare forbid an athletic surgeon to knock down an old gentleman, there was a strategic movement to the rear. The sequel was seen in the police court, where both combatants were bound over to keep the peace. The obvious lesson is that the fiery spirits of Harley Street had better approach their respectable residences by avenues other than those which impinge upon Cavendish Square. Some of these latter just now do undoubtedly put a strain upon the temper of fervent patriots.

An invitation which proposes generous treatment and then restricts within inconvenient limits the dates within which acceptance is possible is apt to excite comment on the restrictions rather than on the generosity. This is likely to be the feeling of those who have received an invitation from a certain Northern health resort with the assurance that free bathing and free drinking of the waters are to be had for the asking, not to speak of a reduced hotel tariff. All this sounds very well until you come to the parenthesis, and then appears the qualification. This announces that all these good gifts and generous offers only come into play when the holiday season is either over or has hardly begun. In these circumstances the volume of gratitude will hardly be overwhelming, and it may be that some of the invited guests will prefer hosts who make a less ostentatious display of their generosity. There are some proposals which lose all their grace when a difficult or impossible qualification is attached to them. "Come in *after* dinner" carries its own interpretation. Moreover, that the English spas should be prepared rather to make the most of their proper virtues than to assume a generosity which no one expects of them is a matter of common sense, the need for which is well seen in the article that appears on page 187.

Medical students' wit, though an unfailing quantity, cannot always boast a high level of refinement and good taste. Probably this was the conclusion reached by certain exalted personages who on a recent visit to one of the hospitals found the cubicles adorned with mottoes and read over one of them "The Abode of Love." To the pure, no doubt, all things are pure, but, considering the recent history of this phrase, it was hardly prudent to flaunt it in a hospital ward. A more happy selection was the one which read "Too ill to be nursed," and which was perhaps derived from the story of a celebrated Edinburgh doctor, who in his day was a prominent figure in the literary circles of the Northern capital. Calling one day in a friendly fashion at the house of one of his patients—a lady well known in academic circles—he was met by the message, "Mrs. M.'s compliments, but she is too ill to see the doctor to-day." The medico had scored many times, but the game was against him on this occasion.

The subject of over-prescribing is a burning one both for panel doctors and panel chemists, as, apart from any question of professional pride, there is a financial aspect to it. In some instances doctors have been surcharged by the insurance authorities, but there is now arising some doubt as to the strict legality of the course which has been pursued. Hence there is a rumour that an important medical organisation, which already boasts not a few legal triumphs, is prepared to take the matter up and to fight it out in the Law Courts. In every direction there are discussions and agitations arising out of the Insurance Act, and what was at one time a dignified and reserved profession seems now to be a subject for discussion and examination in all the village councils of the country. The critic is indeed abroad in the land, and often he is anything but enlightened and well-informed; for the most part, too, he proves himself a very "harbitrary gent."

